# *Notes from the Field*: Increase in Coccidioidomycosis — California, 2016

**DOI:** 10.15585/mmwr.mm6631a4

**Published:** 2017-08-11

**Authors:** Gail Sondermeyer Cooksey, Alyssa Nguyen, Kirsten Knutson, Farzaneh Tabnak, Kaitlin Benedict, Orion McCotter, Seema Jain, Duc Vugia

**Affiliations:** ^1^Infectious Diseases Branch, Center for Infectious Diseases, California Department of Public Health; ^2^Mycotic Diseases Branch, National Center for Emerging and Zoonotic Infectious Diseases, CDC.

Coccidioidomycosis, or Valley Fever, is an infectious disease caused by inhalation of *Coccidioides* spp. spores (*1*). This soil-dwelling fungus is endemic in the southwestern United States, with most (97%) U.S. cases reported from Arizona and California (*1*,*2*). Following an incubation period of 1–3 weeks, symptomatic patients most often experience self-limited, influenza-like symptoms, but coccidioidomycosis also can lead to severe pulmonary disease and to rare cases of disseminated disease, including meningitis (*1*). Those at increased risk for severe disease include persons of African or Filipino descent, pregnant women, adults in older age groups, and persons with weakened immune systems (*1*). In 2016, a large increase in coccidioidomycosis incidence was observed in California compared with previous years (*3*). Using data reported by health care providers and laboratories via local health departments to the California Department of Public Health as of May 9, 2017, incidence rates were calculated by estimated year of illness onset as the number of confirmed coccidioidomycosis cases per 100,000 population (*3*). Estimated year of illness onset was extracted from the closest date to the time when symptoms first appeared for each patient. From 1995, when coccidioidomycosis became an individually reportable disease in California, to 2009, annual incidence rates ranged from 1.9 to 8.4 per 100,000, followed by a substantial increase to 11.9 per 100,000 in 2010 and a peak of 13.8 per 100,000 in 2011 (Figure). Annual rates decreased during 2012–2014, but increased in 2016 to 13.7 per 100,000, with 5,372 reported cases, the highest annual number of cases in California recorded to date.

**FIGURE:**
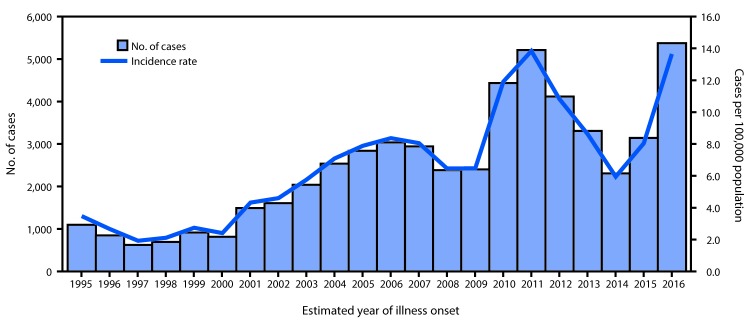
Number of coccidioidomycosis cases and incidence rate, by estimated year of illness onset* — California, 1995–2016 * Estimated year of illness onset was extracted from the closest date to the time when symptoms first appeared for each patient.

Coccidioidomycosis incidence rates increased disproportionately in counties considered to have endemic disease. Most cases in 2016 were in residents of the Central Valley and Central Coast regions, with 42% (2,238 cases, rate 251.7 per 100,000) reported from Kern County and 28% (1,515 cases, rate 54.5 per 100,000) from six other counties (Fresno, Kings, Madera, San Joaquin, San Luis Obispo, and Tulare) (*3*). From 2015 to 2016, the combined incidence from these seven counties increased 109%, from 48.9 per 100,000 (2015) to 102.3 (2016), while the rate in the remaining counties in California increased by 18% (from 3.8 to 4.5 per 100,000).

Reported 2016 incidence was highest among persons aged 40–59 years (18.8 per 100,000), compared with rates in persons aged <20 years (5.6), 20–39 years (14.9), 60–79 years (16.4) and ≥80 years (13.1). However, the sharpest increases in incidence from 2015 to 2016 occurred in persons aged <20 years (134%) and 20–39 years (90%); increases were less pronounced in persons aged 40–59 years (64%), 60–79 years (40%) and ≥80 years (35%). Rates were higher among males (17.3 per 100,000) than among females (10.0). Incidence rates by race and ethnicity were not calculated because these data were missing for approximately one third (32.7%) of reports.

Although annual coccidioidomycosis incidence rates in California and Arizona typically follow similar trends, Arizona reported a decrease in the rate from 2015 to 2016 (from 112.8 to 89.3 per 100,000) (*2*,*4*,*5*). In the remaining states where coccidioidomycosis was reportable in both 2015 and 2016, preliminary data show that incidence remained stable at 0.5 per 100,000 in both years.

The reasons for the increased incidence of coccidioidomycosis in California in 2016, particularly in the Central Valley and Central Coast regions, are not known, but climatic and environmental factors favorable to *Coccidioides* proliferation and airborne release might have contributed, including rainfall after several years of drought and soil disturbance resulting from construction (*2*). To decrease the risk for infection, persons living, working, or traveling in areas where *Coccidioides* is endemic, especially those at increased risk for severe disease, should limit exposure to outdoor dust as much as possible, including staying inside and keeping windows and doors closed during windy weather and dusty conditions (*3*). Previous outbreaks of coccidioidomycosis have occurred among persons working outdoors in areas where *Coccidioides* is endemic, including construction workers; recommendations for reducing the risk for infection on construction worksites include using personal protective respiratory equipment, dust suppression, and worker education (*6*,*7*). Health care providers should be alert for coccidioidomycosis among patients who live in or have traveled to areas where the disease is endemic, especially those who work or participate in activities where dust is generated.
